# Application of Zeolite Tuffs as Mineral Filler in Warm Mix Asphalt

**DOI:** 10.3390/ma13010019

**Published:** 2019-12-19

**Authors:** Agnieszka Woszuk, Michał Wróbel, Wojciech Franus

**Affiliations:** Department of Geotechnics, Faculty of Civil Engineering and Architecture, Lublin University of Technology, Nadbystrzycka 40, 20-618 Lublin, Poland; m.wrobel@pollub.pl

**Keywords:** asphalt, zeolite, clinoptilolite, mineral filler, dynamic viscosity, penetration, softening point

## Abstract

Zeolite materials are used in the warm mix asphalt technology as an asphalt foaming additive, which partially replaces the filler. This article analyzes the influence of the zeolite and other fillers addition on the properties of mastic asphalt. In the research, 35/50 penetration grade asphalt and three types of fillers were used: lime filler (L), zeolite tuff (clinoptilolite) (C), hydrated lime (H) and their mixed combinations (C:L in 1:1 and 1:3 proportions as well as C:L:H in 2:2:1 ratio). The characteristics of the materials included: chemical analysis, phase composition and particle size distribution. The following properties were determined on the asphalt mastic samples: penetration, softening point, penetration index, dynamic viscosity and stiffening measured by softening point increase. It was found that clinoptilolite could partially replace the traditional lime filler, without a negative impact on the asphalt mastic properties. Additionally, the hydrated lime inclusion should have a positive effect on the frost resistance of an asphalt mix.

## 1. Introduction

Asphalt pavements are the dominant type of road surfaces in both Europe and the world. In 2016, the asphalt mix production in the European countries amounted to around 285.5 million tonnes, while in the United States it reached 374.9 million tonnes [1,2]. In addition to mineral aggregates in the asphalt mixture, the filler is one of the major components of the asphalt mixture, which constitutes from a few to several percent. The filler and asphalt mixed together form the so-called asphalt mastic, the properties of which significantly affect the adhesion of mineral aggregates and asphalt as well as the performance of final asphalt mix [3,4,5]. The use of the right type and amount of filler improves the asphalt pavement properties such as stability, durability or even skid resistance. An insufficient amount of filling material may result in reduced rutting resistance. In turn, too much filler causes the asphalt mix to become more fragile and susceptible to low-temperature cracking [6].

The physicochemical characteristics of the mastic depend on the type and properties of the virgin materials used. In the case of fillers, significant parameters are: shape and grain size, textual properties, and chemical composition [7,8,9]. The calcium filler—which is obtained by grinding limestone to the required grain size (0.063 mm in Europe and 0.075 mm in the US)—is used most commonly. Other materials that can be applied to asphalt mix as fillers include fly ash [10] coal waste powder [11], brick dust [12], sewage-sludge ash [13] glass powder [14] and others. Zeolites are also among the materials that are used as an additive to mix asphalt and partially replace the filler.

Zeolites are a group of aluminosilicates with unique physicochemical properties, owing to which they find numerous practical applications in chemical technologies, engineering and environmental protection, agriculture and construction.

Over 100 varieties of different zeolite mineral types are known in nature. Clinoptilolite is the most common one and also most often used in the industry. It crystallizes in a monoclinic system of C2/m space group and unit cell parameters of: a = 1.766 nm; b = 1.726 nm; c = 0.720 nm and β = 116.4°. The most frequently used crystallographic formula of clinoptilolite is in the form of (K_2_,Na,Ca)_3_[Al_6_Si_30_O_72_]·24H_2_O. It is characterized by the occurrence of tetrahedral layers separated by a channel system. The windows of these channels are octagon-shaped rings of 0.44 nm × 0.55 nm in diameter and 10-side rings of 0.44 nm × 0.72 nm in diameter [15].

The zeolite materials are used as a water carrier in warm mix asphalt production to induce the foaming effect [16,17]. The addition of silica gel produces the same effect [18].

Reducing both production and compaction temperatures is the main benefit of using zeolite materials in the asphalt mix. On the other hand, the beneficial effects of lowering technological temperatures are: asphalt mix production costs reduction, lower emissions of fumes, aerosols and hazardous compounds into the atmosphere, improvement of working conditions of production employees and incorporation of warm mix asphalt into the road surface and others. According to the recommendations concerning the principles of designing warm mix asphalt with zeolites, the amount of dosed filler should be reduced proportionally by the amount of the zeolite added [19]. Another possibility is to include zeolite as an "additive", the use of which will not affect the amount of dosed filler.

The previous studies indicate that the mixes produced in the foamed asphalt technology by the addition of zeolites are characterized by the fatigue resistance comparable to hot mixes and a slightly lower stiffness modulus [20,21]. Some studies also indicate the reduced water and frost resistance [22,23,24], especially when using the zeolites soaked with water, which improves the foaming effect of asphalt [25,26]. Resistance to water is improved in the case of WMA with zeolite by the addition of hydrated lime [27,28], which is a commonly used antistripping mineral additive in asphalt pavements. High efficiency in controlling water sensitivity is due to the chemically active nature of this material [29]. Hydrated lime has also been tested for use in asphalt mix as a filler [30,31].

Although according to current design rules, zeolite partially replaces filler [19], the properties of zeolites with regard to their use as fillers have not been tested.

Lack of literature data on this subject motivated the authors to conduct laboratory tests that would allow answering the question of whether zeolite in warm mix asphalt should be treated as a filler or as an independent additive to the asphalt mix. Additionally, the research includes hydrated lime, which can also be used as a filler, while improving the water and frost resistance of an asphalt mix.

## 2. Experimental Materials

The zeolite material was obtained from the clinoptilolite tuffs deposit of Sokyrnytsya, Zakarpattia Oblast, Ukraine. The quantitative content of clinoptilolite in the rock was about 75%. The mineral composition is supplemented with opal CT, quartz and feldspars [32].

The limestone was acquired from Lhoist Bukowa Sp z o.o. (Bukowa, Poland). The limestone was composed of calcite, accompanied by trace amounts of dolomites, clay minerals and quartz [22].

Hydrated lime occurring under the trade name “CX-Z100 hydrated lime” used in the research was provided by CEMEX Polska Sp. z o.o. (Warsaw, Poland). The material is produced by hydrating high-quality active quicklime. It is characterized by very high fragmentation and a large specific surface.

The properties of the materials used in the filler function are presented in Table 1.

The bitumen used in the tests was 35/50 penetration grade asphalt from ORLEN Asfalt Sp. z o.o. (Plock, Poland).The properties of asphalt binder are presented in Table 2.

The 35/50 asphalt is widely used in asphalt mixes incorporated in road pavements in Poland. This type of bitumen is used in base layers, bonding layers and also wearing courses.

## 3. Research Method

### 3.1. Characterization of Clinoptilolite and Limestone

The chemical analysis was carried out using the energy dispersive X-ray fluorescence method (EDXRF) on the Epsilon 3 spectrometer device with the following parameters: RTG Rh 9W, 50 kV, and 1 mA lamp (Panalytical, Almelo, The Netherlands). The analysis included the range of elements from Na to Am. The samples were air-dried.

The laser diffraction method (Mastersizer 3000 with Hydro G dispersion unit with the measuring range of 0.02–2000 μm) was used to determine the particle size distribution (PSD). PSD was determined by the Mie theory application. The following parameters were used: 1.52 for light refractive index along with 0.1 for the absorption coefficient. Briefly, the sample was added to the stirred distillate water to achieve suitable obscuration (10%–20%). The obscuration is a measure of the percentage of laser light that is lost due to scattering or absorption on particles. After adding the sample to the solvent (water in this case) the ultrasounds were used to prepare the stable colloidal solution. The measurements were performed with the pump and stirrer speeds of 1750 and 700 rpm, respectively.

The phase composition of the samples was determined with the X-ray diffraction method (XRD) using X’pert PROMPD diffractometer with PW 3050/60 goniometer, Cu tube and graphite monochromator. The analysis was carried out with an angle ranging from 5 to 65° (2*θ*). The method allows us to determine interstitial spacing d_hkl_ feature for a particular crystal structure according to the basis of Bragg’s law.

### 3.2. Asphalt Mastics Properties

The sample preparation was described according to the methodology used for testing bitumens foamed by the addition of zeolites [16]. Asphalt was heated in the oven to the temperature of 160 °C and then the filler was added into the hot binder and mixed thoroughly for 1 min. The article was supplemented with a description of the methodology for sample preparation.

The softening point was determined according to PN-EN 1427:2009. It is the temperature of asphalt at which the sample located in a standardized ring is heated under certain conditions and then overcomes a certain vertical distance (25.0 ± 0.4 mm) under the weight of standardized metal ball. The parameter was determined in a water test at the initial temperature of 5 °C, which was afterward heated at a rate of 5 °C per minute. In accordance with the technical record, the final result of the test was counted as an average of two measurements rounded to 0.2 °C.

The penetration tests were carried out according to PN-EN 1426:2009. Asphalt penetration was expressed by the depth at which the standardized needle is immersed into the asphalt sample under the weight of 100 g in 5 s at the temperature of 25 °C. The final result of the test was an average of three partial measurements performed on each sample.

The penetration and softening point test results were the basis for determining the penetration index (PI), calculated according to the following formula:

(1) $$\mathrm{PI}=\frac{20\times T_{R\&B}+500\times \mathrm{lgP}-1952}{T_{R\&B}-50\mathrm{lgP}+120},$$

where: T_R&B_—softening point, °C and P—penetration determined in 25 °C, 0,1 mm.

According to the PN-EN 13043:2004 standard, the measure of the stiffening properties of the filler is the increase in the softening point Δ_R&B_ of the asphalt mastic in relation to T_R&B_ of basic 70/100 binder. The test was carried out in accordance with the PN-EN 13179–1:2013 standard.

The dynamic viscosity tests were performed using a Brookfield’s viscometer according to ASTM D 4402, at a temperature of 160 °C. The test consists of calculating the ratio of shear stress caused by rotating spindle to its rotational speed. The viscosity measurements were taken at the specified time intervals: 15, 30 and 45 min, counted from the moment of sample placement in the measuring device. The materials were added to the bitumens in the amount of 5% in relation to mass. Each test was performed on a separate sample. The final result is the average of three partial measurements.

### 3.3. Determination of Samples

The type of the tested material is marked with the following symbols:

35/50—basic 35/50 asphalt,
C—35/50 asphalt with 5% zeolite tuff (clinoptilolite),
L—35/50 asphalt with 5% lime filler,
H—35/50 asphalt with 5% hydrated lime,
0.5C + 0.5L—35/50 asphalt with 5% mixture consisting of 50% zeolite tuff and 50% lime filler,
0.25C + 0.75L—35/50 asphalt with 5% mixture consisting of 25% zeolite tuff and 75% lime filler,
0.4C + 0.4L + 0.2H—35/50 asphalt with 5% mixture consisting of 40% zeolite tuff, 40% lime filler and 20% hydrated lime.

## 4. Results and Discussion

### 4.1. Characteristics of Zeolite Tuff, Limestone and Hydrated Lime

#### 4.1.1. Chemical Composition

The chemical composition of the mineral resources forming the filler is presented in the Table 3. The dominant chemical components of the zeolite tuff were SiO_2_ and Al_2_O_3_. The total content of these ingredients was 75.2%. CaO, K_2_O and Fe_2_O_3_ were present in small amounts, 2.95%, 2.79% and 1.69%, respectively. Other components are found in the trace amounts. In the composition of lime filler and hydrated lime, CaO is definitely dominant, with contents of 74.68% and 97.16%.

#### 4.1.2. Mineral Composition

**Figure 1:**
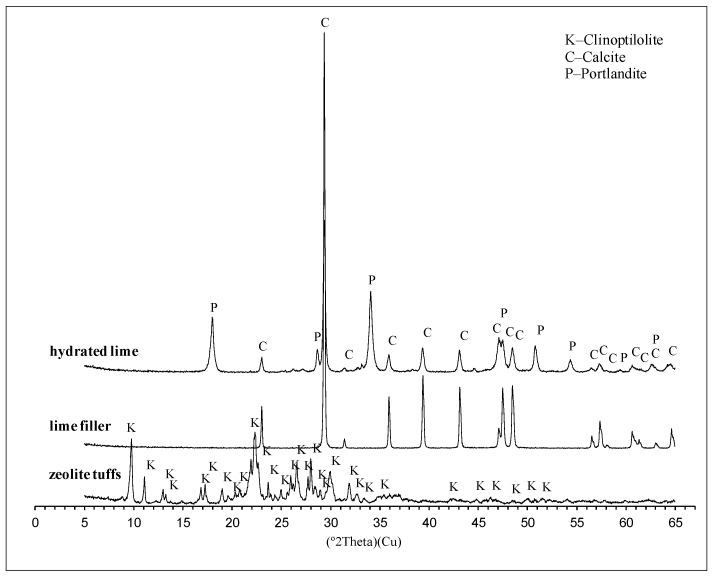
XRD patterns of the tested fillers.

The mineral composition of raw materials determined with the X-ray diffraction method is presented in Figure 1. In the zeolite tuff, clinoptilolite, identified by the interplanar distances d_hkl_ = 0.397, 0.900 and 0.794 nm, was the dominant mineral. Its quantitative content was 78.4%. The other mineral phases were quartz (0.334 and 0.425 nm), cristobalite (0.415 and 0.253 nm), illite (0.100, 0.334 and 0.500 nm) and orthoclase (0.331 and 0.377 nm). The mineral composition of limestone powder contains calcite (99.5%) recognized by distances d_hkl_ = 0.304 and 0.228 nm. In turn, portlandite was recognized in hydrated lime by distance d_hkl_ = 0.262, 0.490 and 0.193 nm, in addition to calcite. The quantitative content of portlandite was 75.7% and 24.3% for calcite.

#### 4.1.3. Grading

**Figure 2:**
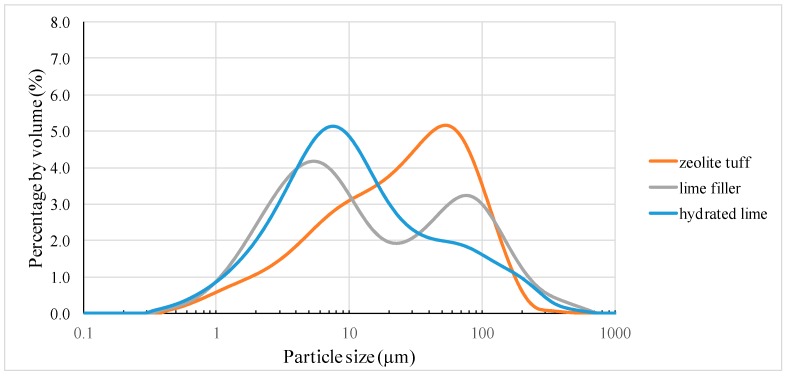
Grading curves of the tested fillers.

The grading curves of tested fillers are presented in Figure 2. All the materials were characterized by bimodal particle size distribution. It is most clearly visible in the case of the limestone filler in which grain sizes of 5 µm and 80 µm dominate. In the zeolite tuff, the grains with a size of 55 µm, accompanied by grains with a size of 0.9 µm constitute the majority. For hydrated lime, grain sizes of 8 µm and 80 µm were dominant.

Petersen et al. stated that the lime–bitumen interaction, improving the asphalt mix water and frost resistance, may occur because of an increase in specific surface, the nature of the Ca(OH)_2_, or a combination of both [33]. It was also proven that the fillers with the presence of strong bases like Ca(OH)_2_, CaO and Mg(OH)_2_·Ca(OH)_2_ reduce the aging of bitumen [34]. B. Huang et al. also found that the asphalt mix with the addition of very fine-grained hydrated lime turned out to be more frost resistant [35].

### 4.2. Asphalt Mastics Properties

**Figure 3:**
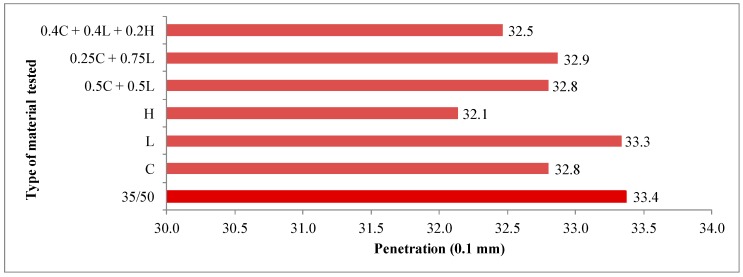
The results of penetration tests for 35/50 asphalt and asphalt mastics containing fillers addition.

**Figure 4:**
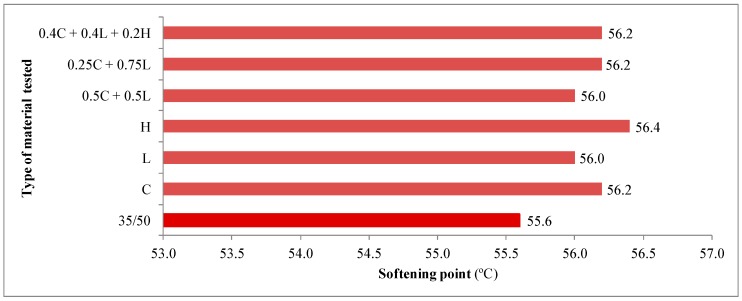
Results of softening point tests for 35/50 asphalt and asphalt mastics containing fillers addition.

**Figure 5:**
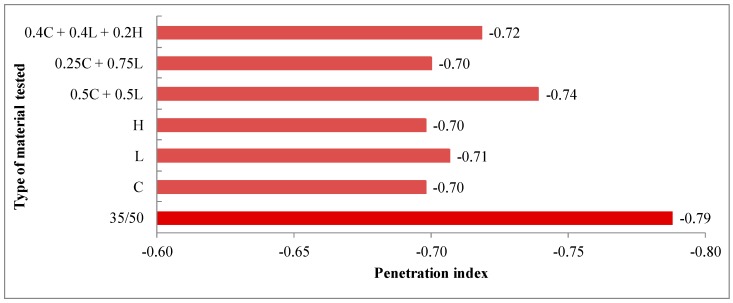
Penetration index of 35/50 asphalt and asphalt mastics containing fillers addition.

**Figure 6:**
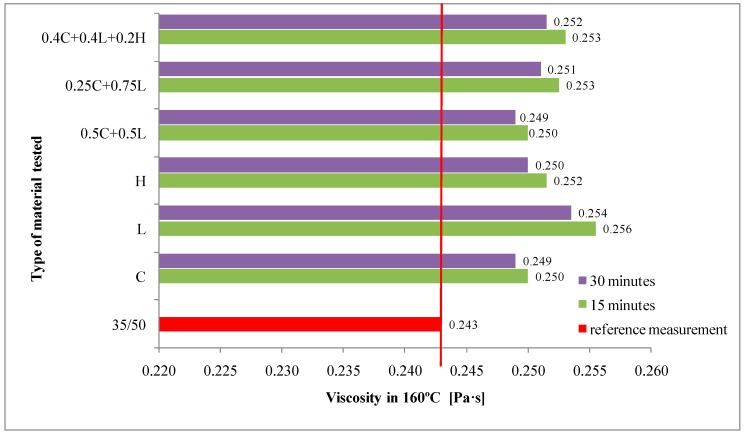
The results of dynamic viscosity tests for 35/50 asphalt and asphalt mastics containing fillers addition.

**Figure 7:**
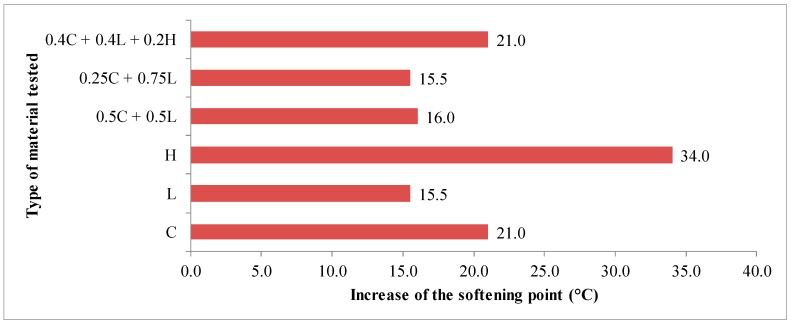
The results of stiffening properties tests for 70/100 asphalt and asphalt mastics containing fillers addition determined by softening point increase Δ_R&B_.

The average results of 35/50 asphalt tests as well as asphalt mastics are presented in Figure 3, Figure 4, Figure 5, Figure 6 and Figure 7.

The penetration of the 35/50 asphalt was 33.4 × 0.1 mm. The addition of the fillers caused a slight decrease in the parameter, which is consistent with the test results of the bitumens with Na-P1 zeolite addition [25]. As noted by Nciri et al., this phenomenon is caused by the stiffening of asphalt binder after mixing with dust fraction. It consists of the following processes [6]:

(1) The stiffening caused by the presence of rigid inclusions;

(2) The stiffening caused by the interfacial effects between filler particles and asphalt;

(3) Particle-interaction reinforcement.

A correlation between the change in asphalt penetration and the specific surface of the used filler was observed: the higher the specific surface, the greater decrease in the studied parameter. When lime filler was used, the smallest decrease in penetration was noted—by 0.1 × 0.1 mm, while in the case of hydrated lime addition—by 1.3 × 0.1 mm. The above-mentioned relationship was also maintained for mixed fillers: limestone + clinoptilolite and limestone + clinoptilolite + hydrated lime.

The softening point of the 35/50 asphalt was 55.6 °C, whereas the parameter determined on asphalt mastics depended on the type of the filler used and equaled: 56.0 °C for lime filler, 56.2 °C for clinoptilolite as well as 56.4 °C for hydrated lime. The change in softening point, as in penetration case also depends on the texture properties of the added filler. The larger the specific surface area, the greater the increase in the tested parameter. With the mixed filler addition, an increase in the softening point was also observed as well.

Xu et al. reported that in the asphalt mastics with fine limestone powder and coal waste ash additives, the dynamics of changes in penetration and softening point depends on the density and volume density of the used materials [36].

Based on the results of penetration and softening point tests, the penetration index was calculated, which amounted to −0.79 for the 35/50 asphalt. The analysis of the parameter indicates a slight decrease in thermal sensitivity of asphalt after using the additives. The optimal value of penetration index, which ranged from −1.0 to 1.0 for road bitumens, was maintained for all types of fillers.

Obtained research results illustrate that the types of fillers used in an amount of 5% in relation to the mass of asphalt did not significantly affect the physical properties of the tested asphalt such as penetration, softening point and thermal sensitivity. Increasing the amount of filler, a wider range of changes could be expected, as shown by studies conducted by Nciri with oyster shell powder (OSP) in the amounts of 5%, 10% and 15% [6].

The results of dynamic viscosity tests for basic bitumen and asphalt mastic measured in 160 °C are presented in Figure 6.

The viscosity of 35/50 asphalt was 0.243 Pa·s, whereas with the addition of the tested fillers it was consistently higher than in the case of the reference sample. This is the effect of insoluble solid insertion into the binder, which does not form a homogeneous liquid together [16]. The highest increase in viscosity was observed with the limestone filler addition. In the case of clinoptilolite and hydrate lime addition, the viscosity of obtained asphalt mastic is similar to each other −0.249 Pa·s and 0.250 Pa·s, respectively. Comparable results were noticed when using limestone-zeolite filler in 1:1 and 3:1 ratio as well as adding mixed filler containing limestone, clinoptilolite and hydrated lime in 2:2:1 ratio. Regardless of the type of dosed filler, a slight decrease in viscosity was observed 30 min after the test beginning (45 min after dosing the additive to the asphalt binder). The same relationship was obtained when using other types of zeolite materials [16,25].

The stiffening properties of the tested materials determined by the increase in Δ_R&B_ softening point of the asphalt mastic are presented in Figure 7.

On the basis of the obtained test results, it was found that the limestone filler had a lower stiffening effect (15 °C) than clinoptilolite (21 °C). This was due to the higher porosity of the zeolite material. In reference to this fact, it could be concluded that the zeolite tuff had slightly worse stiffening properties than the limestone filler. The use of mixed limestone-zeolite filler in 1:1 or 1:3 ratios resulted in a slight change in the increase of softening point by no more than 1 °C. The greatest stiffening effect was observed in the case of hydrated lime (34 °C). For this type of material, the increase of the stiffening point was more than twice as high when using conventional limestone. The asphalt mastic with the addition of limestone and limestone–zeolite filler in the 1:1 or 1:3 proportion was characterized by a small increase in softening point in the Δ_R&B_ category 8/16. The use of clinoptilolite or limestone-clinoptilolite-hydrated lime mixed filler in the 2:2:1 ratio caused the growth of Δ_R&B_ category to 8/25. According to the current technical regulations in Poland [37], the filler of these categories can be used in all types of asphalt mixes. In the case of hydrated lime, due to the high stiffening of the asphalt mastic, it cannot be used as an independent filler.

As noted by V. Antunes et al., very high stiffening power is the effect of geometric characteristics of this material (granulous and rough particles with a tendency to agglomeration) [38]. Furthermore, it is worth mentioning that the inclusion of hydrated lime filler particles toughens the mastic, making it more resistant to fracture and crack propagation [29].

### 4.3. Statistical Data Analysis

A one way ANOVA test was used for statistical analysis of penetration, softening point and viscosity results. It is a parametric tool allowing the comparison of more than two groups separated by the categories of one variable. This method is an extension of the Student’s *t*-test for comparison between two independent groups.

The results of the analysis presented in Table 4 indicate that the effect of the filler type was statistically insignificant for the penetration results (*p* = 0.05553 > 0.05) and softening point (*p* = 0.56303 > 0.05).

In the case of viscosity tests, the use of 5% filler addition was statistically significant. Based on a one-way analysis of variance, it is not possible to determine which type of filler was applied. For this purpose, multiple (post hoc) comparison tests were performed using the LSD method (least significant difference). This method consists of performing a series of *t*-tests for each comparable pair of average results.

The analysis presented in Table 5 consisted of the comparison of the viscosity results obtained for asphalt mastics to the viscosity of bitumen 35/50.

The statistical analysis shows that the addition of filler regardless of the type had a statistically significant effect on the viscosity of the 35/50 asphalt. For all filler types, the obtained value of p coefficient was from 0.00304 to 0.01243 and was much lower than the assumed value of p = 0.05.

## 5. Conclusions

The analysis of the test results shows that the addition of lime filler, clinoptilolite or hydrated lime did not significantly affect the properties of tested bitumen such as: consistence, softening point, thermal sensitivity and dynamic viscosity. At the same time, a correlation between the change in penetration and softening point of the asphalt and the specific surface of the used materials was observed.

Considering the stiffening properties of zeolite tuff, just like the limestone, it can be used as a stand-alone filler in an asphalt mix, despite the greater bitumen hardening. In the case of hydrated lime, it was observed that its addition caused more than a double increase in softening point compared to limestone filler and did not meet the technical requirements.

The research results of mixed filler additive (C:L in 1:1 and 1:3 ratio) indicate that zeolite could partially replace the filler in asphalt mix without a negative impact on the asphalt binder properties.

In addition, these results indicate that the procedure of designing a warm mix of asphalt, where zeolite was considered as a part of the filler was correct. However, the inclusion of zeolite as a separate “additive” in the recipe of asphalt mix might result in a too high increase in the dust fraction, which in consequence might harm the properties of mastic asphalt and asphalt mix.

Hydrated lime has a large specific surface area and a strong alkaline character. Therefore, its addition to an asphalt mix should improve the frost resistance. The analysis of the test results indicates the possibility of using mixed filler C:L: H in asphalt mixed in a 2:2:1 ratio.

In order to verify the preliminary results of the tests, further research on the physical and mechanical properties of asphalt mix is required.

## Figures and Tables

**Table 1:** Of the tested fillers.

Test	Specification	Result		
		Zeolite Tuff	Limestone	Hydrated Lime
Particle density (g/cm^3^)	EN 1097-7:2008	2.256	2.763	2.441
Specific surface area (cm^2^/g)	EN 196-6:2011	10338	6783	12532
Grading (mm)	EN 933-10:2009	0.0–1.0	0.0–1.0	0.0–0.25
Water content (%)	EN 1097-5:2008	<1.0	<1.0	<1.0

**Table 2:** Of the basic bitumen.

Test	Specification	Result
Penetration (25 °C, 0.1 mm)	EN 1426:2009	33.4
Viscosity at 135 °C (mPa s)	ASTM D 4402	748
Viscosity at 160 °C (mPa s)	ASTM D 4402	243
Softening point (°C)	EN 1427:2009	55.6

**Table 3:** Chemical composition of the tested fillers.

	Zeolite Tuff	Lime Filler	Hydrated Lime
	(% of weight)		
Na_2_O	0.01	0.00	0.00
MgO	0.77	0.21	0.33
Al_2_O_3_	9.28	0.15	0.45
SiO_2_	65.92	0.27	0.90
P_2_O_5_	0.03	0.02	0.00
SO_3_	0.01	0.04	0.40
K_2_O	2.79	0.00	0.09
CaO	2.95	74.68	97.16
TiO_2_	0.23	0.00	0.00
Fe_2_O_3_	1.69	0.06	0.62

**Table 4:** ANOVA analysis on the parameters of the 35/50 asphalt and asphalt mastics containing fillers addition.

Test Type	Origin	SS	df	MS	F	*p* value	Test F
Penetration	Intergroups	3.484762	6	0.580794	2.753198	0.055535	2.847726
	Intragroups	2.953333	14	0.210952			
Softening point	Intergroups	0.758571	6	0.126429	0.863415	0.563036	3.865969
	Intragroups	1.025	7	0.146429			
Dynamic viscosity	Intergroups	0.000194	6	3.24E-05	12.37576	6.75E-05	2.847726
	Intragroups	3.67E-05	14	2.62E-06			

**Table 5:** Result of the multiple comparison test by the method of least essential differ dynamic viscosity asphalt mastics containing fillers addition.

Type of Used Filler	*p* value
C	0.00873
L	0.00676
H	0.01243
0.5C + 0.5L	0.01015
0.25C + 0.75L	0.00743
0.4C + 0.4L + 0.2H	0.00304
